# Investigating Predictive Factors of Suicidal Re-attempts in Adolescents and Young Adults After a First Suicide Attempt, a Prospective Cohort Study. Study Protocol of the SURAYA Project

**DOI:** 10.3389/fpsyt.2022.916640

**Published:** 2022-06-29

**Authors:** Erika Abrial, Benoît Chalancon, Edouard Leaune, Jérôme Brunelin, Martine Wallon, Frédéric Moll, Nadine Barakat, Benoit Hoestlandt, Anthony Fourier, Louis Simon, Charline Magnin, Marianne Hermand, Emmanuel Poulet

**Affiliations:** ^1^Centre Hospitalier Le Vinatier, Bron, France; ^2^INSERM U1028, CNRS UMR5292 Lyon Neuroscience Research Center, PSYR2 Team, Bron, France; ^3^Lyon 1 University, Villeurbanne, France; ^4^INSERM U1028, CNRS UMR5292 Lyon Neuroscience Research Center, WAKING Team, Bron, France; ^5^University Hospital Edouard Herriot, Hospices Civils de Lyon, Lyon, France; ^6^Laboratory of Medical Biology and Anatomo-Pathology, Hospices Civils de Lyon, Groupement Hospitalier Est, Bron, France; ^7^Louis-Mourier Hospital, Assistance Publique Hôpitaux de Paris, Colombes, France

**Keywords:** young adults, cortisol, Brain-Derived Neurotrophic Factor, inflammation, toxoplasmosis, predicting, suicide attempt

## Abstract

**Introduction:**

Suicide is the fourth leading cause of death in youth. Previous suicide attempts are among the strongest predictors of future suicide re-attempt. However, the lack of data and understanding of suicidal re-attempt behaviors in this population makes suicide risk assessment complex and challenging in clinical practice. The primary objective of this study is to determine the rate of suicide re-attempts in youth admitted to the emergency department after a first suicide attempt. The secondary objectives are to explore the clinical, socio-demographic, and biological risk factors that may be associated with re-attempted suicide in adolescents and young adults.

**Methods:**

We have developed a single-center prospective and naturalistic study that will follow a cohort of 200 young people aged 16 to 25 years admitted for a first suicide attempt to the emergency department of Lyon, France. The primary outcome measure will be the incidence rate of new suicide attempts during 3 months of follow-up. Secondary outcomes to investigate predictors of suicide attempts will include several socio-demographic, clinical and biological assessments: blood and hair cortisol levels, plasma pro- and mature Brain-Derived Neurotrophic Factor (BDNF) isoforms proportion, previous infection with *toxoplasma gondii*, and C-Reactive Protein (CRP), orosomucoid, fibrinogen, interleukin (IL)-6 inflammatory markers.

**Discussion:**

To our knowledge, the present study is the first prospective study specifically designed to assess the risk of re-attempting suicide and to investigate the multidimensional predictive factors associated with re-attempting suicide in youth after a first suicide attempt. The results of this study will provide a unique opportunity to better understand whether youth are an at-risk group for suicide re-attempts, and will help us identify predictive factors of suicide re-attempt risk that could be translated into clinical settings to improve psychiatric care in this population.

**Clinical Trial Registration:**

ClinicalTrials.gov, identifier: NCT03538197, first registered on 05/29/2018. The first patient was enrolled 05/22/2018.

## Introduction

Suicide is a major public health concern, causing more than 700,000 deaths per year worldwide (1). Moreover, as suicidal behaviors prevalence increases during adolescence (2), suicide is reported to be the fourth leading cause of death among people aged 15 to 29 (1). Having a history of suicide attempts is the most important risk factor for suicide, with a recent study reporting a higher risk of suicide mortality for 6 months following a suicide attempt, with a peak mortality risk at 1 month (3). Similarly, the risk of suicide re-attempt is at its highest in the first 6 months after an index attempt, and declines over time (4). The dangerousness of the means and its lethality increase with age (5). With fewer studies focusing on adolescents and young adults, suicide re-attempts are reported to occur in this population in 12% at 3 months (6), 17% at 6 months (7), and in 25–31% at 1 year (8).

The biopsychosocial model of suicide theorizes suicidal behaviors as a result of complex trait and triggering factors including socio-demographic, clinical, and biological determinants (9). Identified individual risk factors for suicide and suicide re-attempts include: a diagnosed psychiatric disease, family history of suicide and psychiatric illness, substance abuse, impulsivity, sexual or physical abuse and bullying, active suicidal ideation, conflicts with romantic relationship, and, most importantly, a personal history of suicide attempt (10).

Regarding the biological underpinnings, most studies conducted in adults have shown that suicidal behaviors could be associated with lower baseline, chronic, and/or reactivity of cortisol levels (11–13), suggesting a failure of the hypothalamo-pituitary-adrenal (HPA) axis stress-response system. Other biological markers of interest involve proinflammatory markers such as C-Reactive Protein (CRP) and interleukin (IL)-6 (14–16), a history of exposure and seropositivity to *toxoplasma gondii* (17), and low levels of Brain-Derived Neurotrophic Factor (BDNF) (18, 19), although the evidence to support their implication is less consistent (20, 21).

These biological processes may be particularly relevant during the transition from adolescence to adulthood, a critical period of development characterized by exposure to interpersonally-themed stressors, increased impulsivity and risk-taking behaviors, and onset of severe psychiatric disorders (22).

Altogether and despite the known burden of youth suicide, this population has been poorly investigated and there is scarce knowledge regarding the potential factors that may differentiate those who will make repeated attempts from those who will not. Therefore, this study is aimed to define the incidence of suicide re-attempt in young first-attempters, and to investigate the association between socio-demographic, clinical, and biological (HPA axis activity with blood and hair cortisol, pro-inflammatory markers with CRP, fibrinogen, orosomucoid and IL-6, infection with *toxoplasma gondii*, and neuroplasticity imbalance measured by the proportion of mature, and pro-BDNF isoforms) variables. Given their specific characteristics, we hypothesize that young first-attempters would be at higher risk of early suicide re-attempt compared to adults.

## Methods

The present prospective cohort SURAYA (SUicide Re Attempts in Young Adults) is currently underway in Lyon (France) and is expected to end in October 2022. The study will involve two investigation centers: recruitment and baseline data collection will take place in the Psychiatric Crisis Unit of Edouard Herriot Hospital (Hospices Civils de Lyon, Lyon, France), and the primary outcome and data at 3 months in the Centre de Prévention du Suicide (*Centre Hospitalier le Vinatier, Bron, France*). The sponsor of the study is the “*Centre Hospitalier le Vinatier*,” Bron, France. The study is conducted in accordance with the recommendations provided in the current version of the Declaration of Helsinki. This study was approved by the local ethics committee (Comité de protection des personnes Sud Méditerranée III—on 01/02/2018) and by the National Agency for the Safety of Medicines and Health Products (ANSM registration number 2017-A03129-44). The study was preregistered in a public database, first registered on 5 May 2018 (https://clinicaltrials.gov registration number: NCT03538197).

### Participants

The inclusion criteria are: (1) be hospitalized for a first suicide attempt in an emergency psychiatric unit, (2) be between 16 and 25 years old, (3) speak fluent French. Participants under curator- or guardianship were not eligible. Interrupted, but not aborted, suicide attempts will also be included. Medical records, when available, will be consulted to avoid errors of inclusion. We will also use the “lifetime suicidal behaviors” section of the C-SSRS to detect false inclusions. All participants will be required to provide written informed consent after a full and fair description of the objectives and needs of the study. Consent for minors will be obtained from the participant and at least from one parent as legal representative.

### Study Design

**Figure 1:**
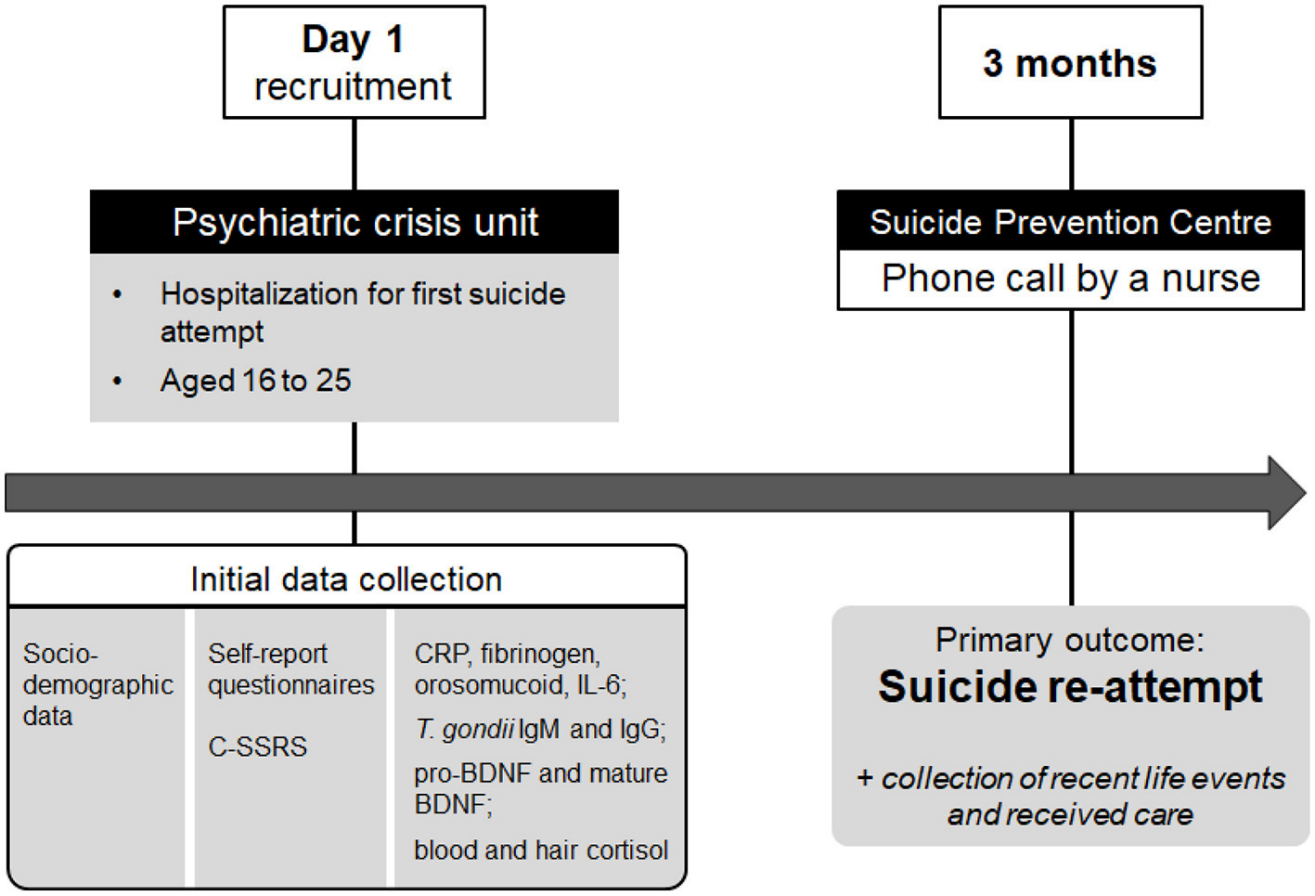
Study design. On day 1, first-time suicide attempters aged 16 to 25 will be included in the SURAYA prospective cohort study upon their hospitalization in a psychiatric crisis unit. During their stay, socio-demographic, clinical and biological data will be collected. Three months later, participants will be contacted to retrieve the primary outcome: the presence or absence of a second suicide attempt *(re-attempt)* within the first 3 months. *C-SSRS, Columbia Suicide Severity Rating Scale; CRP, C-Reactive Protein; BDNF, Brain-Derived Neurotrophic Factor; Ig, immunoglobulin; IL-6, interleukin-6; SURAYA, SUicide Re Attempts in Young Adults*.

The overall study design is shown in Figure 1. According to our sample size calculation (see below), 200 adolescents and young adults will be recruited in the study. Upon enrollment, baseline data will be collected. Clinical and socio-demographic data will be investigated through the computerized clinical chart and through relatives if necessary. Psychiatric diagnoses will be established according to the Diagnostic and Statistical Manual of Mental Disorders, Fifth edition (DSM-5) during a standardized psychiatric interview. During hospitalization, participants will complete standardized and validated questionnaires. The morning after admission to the emergency unit, a first blood sample will be taken from fasting patients at 7:00 am for biological measurements. Two other blood samples will be collected and stored at −80°C for future research purposes (one EDTA sample and one PAXgene blood RNA tube). Patients who are not fasting will be rescheduled. All inpatients will benefit from usual psychiatric care and will receive appropriate referrals upon discharge. They will also be integrated into the VigilanS brief contact intervention program (23), which is currently being rolled out nationally, and has demonstrated efficacy in reducing suicide re-attempts (24).

### Baseline Measures

#### Socio-Demographic Factors

The following information will be collected: age, gender, marital and occupational status, education level, socio-economic status, urbanicity level, migratory status, psychosocial issues (including conflicts with parents, access to healthcare), potential traumatic events, and exposure to suicide in the last 6 months.

#### Psychometric Measures and Clinical Factors

Personal and family medical history, current psychiatric diagnosis and treatments, method of attempted suicide will be collected. Psychometric assessments will be performed using a detailed assessment form containing a battery of validated self-administered questionnaires in their French translation. The following dimensions will be documented: depressive symptoms severity with the Quick Inventory of Depressive Symptomatology (QIDS-SR16) (25), risk for bipolar disorder with the Mood Disorder Questionnaire (MDQ) (26), risk for psychosis with the Prodromal Questionnaire-Brief (PQ-B) (27), hazardous alcohol consumption with the Alcohol Use Disorders Test (AUDIT) (28), nicotine dependence with the Fagerstrom Questionnaire (29), cannabis abuse with the Cannabis Abuse Screening Test (CAST) (30) and the Cannabis Use Disorders Identification Test, revised (CUDIT-R) (31), impulsiveness with Barratt Impulsiveness Scale (BIS-10) (32), anger with the State-Trait Anger Expression Inventory 2 (STAXI-2) (33), childhood traumatic experiences with the Childhood Trauma Questionnaire (CTQ) (34), protective factors against suicidal behaviors with the Reasons For Living (RFL) inventory (35).

Moreover, patients will complete non-validated self-reports: visual analog scales for subjective moral pain, physical pain, suicidal ideation intensity, and a 12-item questionnaire on negative life events during the last 6 months.

The Columbia Suicide Severity Rating Scale (C-SSRS) will be administered by a trained clinician. The C-SSRS is a semi-structured interview with solid psychometric properties designed to assess grading of suicidal ideation and behaviors, validated in various general and clinical populations, including adolescents and adults presenting for psychiatric care (36). In a recent study by Lindh et al. (37), C-SSRS appears to perform well in predicting suicide attempts within 3 months, compared to other suicide risk scales.

#### Biological Factors

The levels of CRP, orosomucoid, fibrinogen, cortisol, IL-6, and anti-toxoplasma gondii immunoglobulin G (IgG) and immunoglobulin M (IgM) will be measured in the blood as routine analyses by the central biological laboratory of the hospital.

To assess a potential imbalance between neurotrophic and proapoptotic systems (38), we will measure the proportion of mature BDNF and pro-BDNF respectively, as exploratory analyses. Plasma mature BDNF and pro-BDNF levels will be assessed by enzyme-linked immunosorbent assay (Biosensis, BEK-2211/2237) in collaboration with biologists from Hospices Civils de Lyon. As a measure of chronic stress exposure in contrast to blood cortisol analysis, we will measure hair cortisol levels. Hair strands will be cut carefully with scissors as close as possible to the scalp, and stored at room temperature in aluminum foil. Cortisol levels will be determined from the 3 cm segment of hair closest to the scalp. This represents hair growth over the 3-month period prior to sampling based on an average hair growth of 1 cm/month. In collaboration with University Hospital of Bordeaux, assays will be performed by Liquid Chromatography with tandem mass spectrometry as previously detailed in the study of Brossaud and colleagues (2021) (39).

### Outcomes

Three months after the initial hospitalization for a first suicide attempt, participants will be contacted by phone to retrieve the primary outcome: the presence or absence of a second suicide attempt *(re-attempt)* within the first 3 months following the first suicide attempt. The data will be collected by a nurse blinded to the patients' initial characteristics, and will be further controlled by 3 independent reviewers to avoid misclassification. Other information regarding recent life events and received care will be gathered. If the patient cannot be reached, their trusted person, previously designated by the patient, will be contacted. In case of no reply, the primary care physician or medical records will be consulted. A suicide attempt is defined as a non-fatal self-directed potentially injurious behavior with the intent to die as a result of the behavior. Secondary outcomes will be baseline socio-demographic, clinical, and biological factors.

### Statistical Analysis

Statistical analysis will be performed using R software version 4.02. The significance level alpha will be set at 0.05, and all statistical tests will be 2-tailed.

#### Sample Size Calculation

Based on the largest survey study conducted in France by Vuagnat and colleagues (2019) (4), we estimated that 12.4% of the population will be readmitted for suicidal attempt within the 3 months following a first suicide attempt that led to hospitalization. This is also in accordance with the study from Spirito and colleagues (2003) (6) conducted in a comparable sample of youth individuals (12%). Using the Wald Confidence Interval method, we calculated that a sample of 200 participants will be sufficient to reach a precision of +/– 4% calculated as 95 CI = p +/– 1.96 sqrt (p (1–p)/n), with *p* = 0.124.

#### Primary Outcome

First, the classic incidence rate will be calculated by dividing the total number of new re-attempt cases by the total number of suicide attempters. Next, the person-time incidence rate will be calculated by dividing the total number of new re-attempt cases by the sum of the person-time of attempters. This proportion will be compared to the estimated 12.4% based on the literature.

#### Secondary Outcomes

Chi-squared (χ2) tests will be used to assess the relationship between re-attempts and qualitative variables; independent *t*-test and Mann-Whitney will be used to compare parametric and non-parametric variables between the two groups of re-attempts and no re-attempts. Variables with a *P*-value lower than 0.2 will be included in a multiple Cox regression model to estimate the adjusted hazard ratio (AOR) with a 95% confidence interval (CI) for the risk factors associated with re-attempt suicide. In all the tests, the confidence interval will be 95%, and *P* < 0.05 to be considered significant.

## Discussion

The main purpose of the SURAYA study is to assess the incidence of suicide re-attempt after a first attempt in a cohort of young people aged 16 to 25. The study's main strength is the homogeneity of participants: a narrow age group relevant to a critical period of neurodevelopment, and an incipient cohort in terms of onset of suicidal behaviors. There is little research focusing on suicidal behaviors in adolescents and young adults, and we do not currently know whether this group is at a higher risk of early suicide re-attempt compared to a general adult population.

Taken separately, most suicide risk factors previously identified are reported to be weak predictors of later suicidal ideation and behavior. In order to gain a comprehensive view of the potential prognostic factors of suicide re-attempts, we carefully collected a wide range of socio-demographic, clinical, and biological data relevant to suicidality; we used both self- and clinician-administered validated scales for more reliability across psychometric evaluations. We emphasized on emerging psychiatric disorders, addictions, psychotrauma, impulsivity and anger, environmental triggering factors, and severity of the first suicide attempt with the C-SSRS.

Beyond these features, we chose to include participants regardless of their psychiatric diagnosis, to capture transnosographic correlates of suicide re-attempts, and thus minimize the effect of potential confounding factors of comorbid psychiatric disorders. However, ICD10 diagnostic criteria will be included in the analysis of conditions such as depression or borderline personality disorder being particularly prevalent in the included population. Attenuated psychotic symptoms (i.e., ultra-high risk population), measured by the PQ-B, are also an interesting symptomatic dimension, as the onset of the disorder can frequently be manifested by an act of aggression. A recent meta-analysis of suicide risk during this time period suggests that suicidal and self-harming thoughts and behaviors were highly prevalent in the ultra-high risk population (40).

Impulsivity can be described as a general pattern of behavior (trait-impulsivity), as responses that are not conformed to their context (action-impulsivity), or as inability to delay reward or to take future consequences into account (choice-impulsivity). Due to a delayed development of top-down prefrontal areas relative to subcortical regions involved in desire and fear, adolescents and young adults are particularly susceptible to impulsive behaviors (41), which suggests an increased risk of suicidal behaviors. This dimension therefore constitutes an interesting criterion for suicide re-attempt (42).

For biological measures, we will explore whether suicide re-attempt could be associated with alterations of the HPA axis, neuroplasticity, and inflammation, including a recent toxoplasmosis infection. These biological systems are highly connected since glucocorticoids dysregulation could lead to increased inflammatory activity and impaired neuroplasticity (43, 44).

Cortisol is the key hormone of the stress-response system, and failure of the HPA axis in response to stress may underlie suicidal crises (22). Most findings, conducted in the general population, suggest that both lower and higher baseline and stressor-induced cortisol levels have been associated with suicide, consistent with allostatic load theories resulting from the adaptation to the environment (22, 45). Accordingly, we will quantify blood cortisol levels as a measure of an acute stress provoked by the suicide attempt, and, in collaboration with the University Hospital of Bordeaux, we will assess hair cortisol, as a retrospective indicator of cumulative cortisol levels during the last 3 months preceding the attempt. To date, hair cortisol has been used in various settings as a reliable marker of long-term exposure to stress (46–48). A recent meta-analysis investigating the association between hair cortisol levels and depression found contradictory results (49). Nonetheless, because altered functioning of the HPA axis has been strongly involved in major depressive disorder (50), depression is a potential confounding variable of cortisol measures and will be considered in the analysis. Similarly, thyroid dysfunction may result in depressive symptoms (51). Thyroid function tests were not systematically performed in the cohort, thus we cannot rule out depression with subclinical hypothyroidism.

BDNF is able to cross the blood-brain barrier and its plasma levels reflect the central nervous system levels (52). In addition, studies suggest that the mature form of BDNF and its precursor pro-BDNF could have opposite functions on neural plasticity: while mature BDNF promotes neuronal survival and growth, pro-BDNF induces neuron apoptosis (53). It thus appears essential to measure not only total BDNF, which does not reflect this balance between pro-BDNF and mature BDNF.

For the past few years, the immune system has been a growing focus of interest in suicide biomarker research. Particularly, studies have suggested a dysregulation of anti- and pro-inflammatory cytokine balance (54, 55). However, in a recent systematic review (56), the role in suicidal behaviors of central and peripheral interleukins, their genes and polymorphisms, remained inconclusive. The authors underline the heterogeneity of the samples, as well as potential confounding factors of inflammation, which are often not taken into account. As an exploratory measure of peripheral inflammation, we have chosen to measure the levels of IL-6, one of the most studied pro-inflammatory cytokines. Additionally, we will measure fibrinogen, orosomucoid, and CRP, three non-cytokinic inflammatory factors. CRP is an acute-phase inflammatory protein synthesized in response to serum IL-6 increase (57) which has been associated with suicide (15, 16), whereas fibrinogen and orosomucoid are two other acute-phase proteins which has not yet been investigated in suicidal behaviors. For further studies, others peripheral non-cytokinic biomarkers of interest are serum S100B, that have been associated with suicidal ideation and behaviors in adolescents (58), and the renin-angiotensin system, at the interface between inflammation and the HPA axis, with polymorphisms of the angiotensin-I converting enzyme associated with suicide attempts and completions (59). Interestingly, combining different inflammatory markers into an inflammatory index as did O'Donovan et al. (16) could be a valuable approach.

The neuroimmune network hypothesis proposes reciprocal interactions between the immune system and the brain. In addition to activation of the HPA axis, peripheral cytokines are able to reach the brain through humoral, neural, and cellular pathways (60). Functional neuroimaging studies in adults suggest that systemic inflammation is associated with altered resting state functional connectivity (RSFC) within several brain networks associated with cognition and mood regulation (61–63). Notably, Marsland and colleagues (2017) (61) showed that within the default mode network (DMN), higher levels of IL-6 were positively correlated with connectivity of the subgenual anterior cingulate cortex and negatively correlated with the dorsal medial prefrontal cortex. Another study reported that induced inflammatory state was associated with decreased connectivity between salience network regions, including the insula, amygdala, dorsal anterior cingulate cortex, and anterior prefrontal cortex (63). Few studies have examined these associations in youth, yet scarce data point to different networks than adult studies (64, 65). Likewise, little is known about the functional connectivity patterns associated with suicidal behaviors in adolescence and young adulthood, which contrasts with the substantial changes of these RSFC during this unique neurodevelopmental phase (66). Interestingly, recent studies comparing young depressed patients with or without a history of suicide attempt showed reduced connectivity between the anterior DMN and the salience network (67), and decreased RSFC between left prefrontal-right anterior cingulate cortices, the latter being related to higher trait-impulsivity in attempters (68). Long-term alterations of functional connectivity could lead to structural changes in underlying brain areas (69). Of particular interest to our study, one prospective study showed that reduced baseline gray matter volume and white matter integrity in frontal areas differentiated adolescents and young adults with mood disorders who later attempt suicide (70), highlighting the need to combine neuroimaging markers with other biopsychosocial markers in future studies.

When interpreting our results, we will take into account potential limiting factors. First, since all inclusions will take place in Edouard Herriot University Hospital, we expect a center-effect bias. Inpatients of our unit may have more severe disorders and suicidal behaviors. Besides, patients admitted to the unit do not exhibit severe behavioral problems nor are hospitalized without consent, which may exclude a certain group of suicide attempters. This could lead to a lack of representativity and limit the external generalizability of the results. Second, loss to follow-up is common in longitudinal studies and could potentially cause selection bias. In our study, we have not included the risk of drop-outs in the calculation of the number of subjects needed. However, a follow-up period extending over a relatively short period of 3 months could reduce loss to follow-ups. To further limit this bias, we prefer phone calls at 3 months over face-to-face consultations. If the patient could not be reached, we set up a 3-level data retrieval process by gathering information from his/her designated trusted person, contacting his/her primary care physician, and finally by consulting his/her medical records. In further cases of inability to obtain information, we have planned a statistical management of missing data. Third, COVID-19 pandemic occurred during the inclusion phase. Recruitment was slowed down due to fluctuating demand for access to care, and to the reorganization of the Department of Psychiatry in our University Hospital. Psychosocial distress due to the pandemic and its consequences could modify suicidal behaviors (71). In addition, there may be stress and inflammation confounding bias related to the COVID-19 status of some inpatients. From March 2020, every patient underwent a SARS-CoV-2 RT-PCR upon admission and only those who were negative were hospitalized in the unit, thus, there was no COVID-19 positive inclusion in this study. Nonetheless, we cannot rule out the possibility of such patients being included prior to the systematic implementation of COVID-19 testing, when the virus circulation was lower in France, i.e., from approximately November 2019 to February 2020. *Post-hoc* subgroup analyses could be needed to assess the impact of potential COVID-19 infection during this time period. Either way, it would be interesting to compare the repercussions of the COVID-19 pandemic on the onset of suicidal behaviors in a future study.

Since preventive interventions have successfully managed to reduce suicide mortality in the general population (72), we hope that the findings of this study will pave the way for early and targeted interventions in youth.

## Trial Status

The recruitment of patients into the trial began in May 2018 and is scheduled to end in October 2022. Preliminary analyses conducted on a sample of the 73 first patients of the cohort (70.8% females; mean age: 19.72 years, SD 2.44) revealed a re-attempt rate at 3 months of 17.81%, supporting a trend toward a higher risk of suicide re-attempt in this population as compared with the literature on adults.

V7 (12/07/2021): MS5 Addition of several blood tests; update of associated investigators and other study stakeholders, update of scientific collaborators.

V6 (04/22/2021): MS4 18 months extension of the recruitment period of the study, as a consequence of COVID-19.

V5 (03/30/2020): MS3 12 months extension of the recruitment period of the study.

V4 (06/03/2019): MS2 12 months extension of the recruitment period of the study.

V3 (07/26/2018): MS1 modification of the inclusion criteria, extension of the study to minors from 16 years of age, and 6 months extension of the recruitment period of the study.

V2 (02/08/2018): Approval of the initial protocol by the ethics committee.

V1 Initial protocol before submission to the ethics committee.

## Ethics Statement

The studies involving human participants were reviewed and approved by the Comité de protection des personnes Sud Méditerranée III−01/02/2018. Written informed consent to participate in this study was provided by the participant, and the participants' legal guardian for minors.

## Author Contributions

EP and MH conceived and designed the study. AF, BC, BH, CM, EA, FM, LS, MW, and NB collected the data. EA, BC, and JB contributed data or analysis tools. EA, EL, EP, JB, and MH wrote the first draft of the manuscript. All authors contributed to the article and approved the submitted version.

## Funding

The study was funded by the Scientific Research Council from Le Vinatier, Psychiatric Hospital (#CSRK01).

## Conflict of Interest

The authors declare that the research was conducted in the absence of any commercial or financial relationships that could be construed as a potential conflict of interest.

## Publisher's Note

All claims expressed in this article are solely those of the authors and do not necessarily represent those of their affiliated organizations, or those of the publisher, the editors and the reviewers. Any product that may be evaluated in this article, or claim that may be made by its manufacturer, is not guaranteed or endorsed by the publisher.

## Abbreviations

AUDIT: Alcohol Use Disorders Test
BDNF: Brain-Derived Neurotrophic Factor
BIS-10: Barratt Impulsiveness Scale 10
C-SSRS: Columbia-Suicide Severity Rating Scale
CAST: Cannabis Abuse Screening Test
CRP: C-reactive protein
CTQ: Childhood Trauma Questionnaire
CUDIT-R: Cannabis use Disorders Identification Test, revised
DMN: Default Mode Network
DSM-5: Diagnostic and Statistical Manual of Mental Disorders, Fifth edition
HPA: Hypothalamic-pituitary-adrenal
IgG: Immunoglobulin G
IgM: Immunoglobulin M
IL: Interleukin
MDQ: Mood Disorder Questionnaire
PQ-B: Prodromal Questionnaire-Brief
QIDS-SR16: Quick Inventory of Depressive Symptomatology, self-report, 16-item
RFL: Reasons for Living
RSFC: Resting State Functional Connectivity
STAXI-2: State-Trait Anger expression Inventory 2.
